# Is inadequate sleep a potential risk factor for periodontitis?

**DOI:** 10.1371/journal.pone.0234487

**Published:** 2020-06-16

**Authors:** Ahmed A. Alhassani, Mohammad S. Al-Zahrani

**Affiliations:** Department of Periodontics, Faculty of Dentistry, King Abdulaziz University, Jeddah, Saudi Arabia; University Lyon 1 Faculty of Dental Medicine, FRANCE

## Abstract

This study was undertaken to investigate the potential association between sleep duration and periodontitis. The study population consisted of 10,291 individuals who participated in the United States National Health and Nutrition Examination Survey (NHANES) from the 2009–2014 cycles. Sleep duration was categorized into sleep deficient (< 7 hours), sleep adequate (7–8 hours), and sleep excessive (> 8 hours). We used the Center for Disease Control and Prevention (CDC) and the American Academy of Periodontology (AAP) periodontitis case definition. Descriptive statistics and logistic regression models were used for data analyses. The prevalence of periodontitis was 36% higher in individuals who reported sleep deficiency when compared to the sleep adequate group (odds Ratio (OR) = 1.36, 95% confidence interval (CI): 1.23–1.50). Those who reported excessive sleep had 41% higher odds of periodontitis (OR: 1.40, 95% CI: 1.16–1.71). After adjusting for confounding factors, sleep deficient individuals were 19% more likely to have periodontitis when compared to sleep adequate individuals (OR: 1.19, 95% CI: 1.06–1.38). Among sleep excessive individuals, the association was non-significant (OR: 1.16, 95% CI: 0.94–1.43). Sleep deficiency was associated with a higher prevalence of periodontitis in this study population. The association however needs to be confirmed in longitudinal studies.

## Introduction

Periodontitis is one of the most frequent chronic conditions in human adults, affecting more than 42% of US adults 30 years of age or older [1]. It is a leading cause of tooth loss among adults and has been reported to have a harmful impact on general health and wellbeing [2–7]; periodontitis has been associated with diabetes, cardiovascular disease and adverse pregnancy outcomes [3–7]. It is a multifactorial disease that entails complex infectious and immunological interactions [8]. Although bacterial plaque biofilm is an essential factor in development of periodontitis, it is insufficient by itself to initiate the disease process. The interplay between the host response and the periodontopathic bacteria is the key to initiation and progression of periodontal inflammation. In a susceptible host, repeated bacterial insult results in dysregulation of the inflammatory and immune pathways leading to persistent inflammation and tissue destruction of the connective attachment, periodontal ligament and alveolar bone [9]. Several environmental and systemic factors such as smoking, diabetes, stress and obesity have been linked to increased susceptibility to periodontitis [10].

Sleep is a natural physiologic process that has restorative and regulatory functions [11]. Sleep duration was found to have a U-shaped relationship with health outcomes. Sleep deficiency is frequently defined as having less than 6 or 7 hours of sleep, while sleep excess is defined as more than 8 or 9 hours [12]. Both deficient and excessive sleep durations are associated with poorer general health. Increased risk for all-cause mortality, diabetes, obesity, and coronary heart disease have been linked to both short and long sleep durations [12–16]. Sleep deprivation was also shown to impair function of the immune system, and could leads to metabolic and endocrine changes that mimic some of the hallmarks of ageing and obesity, which might explain the increase in the severity of age-related pathologies such as diabetes and hypertension [17–19]. Although insufficient nocturnal sleep has been reported to have a harmful impact on several body organs and systems, more than 30% of the American adults sleep less than the recommended 7 hours a night [20].

A few recent studies had investigated the association between sleep duration and periodontitis with conflicting results, reporting negative, positive and no associations between sleep duration and periodontitis [21–25]. These studies had major shortcomings in the classification of the exposure and/or the outcome. A number of them failed to appreciate the widely recognized non-linear (or U-shaped) relationship between sleep and health outcomes [12–16].The purpose of this investigation therefore was to determine if there is a relation between sleep duration and periodontitis in a large nationally representative US sample.

## Methods

### Study population

We used data from the United States National Health and Nutrition Examination Survey (NHANES), 2009–2014 cycles. NHANES is a stratified multistage probability sample of the noninstitutionalized civilians living in United States’ 50 states and District of Columbia [26]. In NHANES, calibrated examiners who were either registered dental hygienists or general dentists performed the periodontal examination in mobile examination centers for participants who were ≥ 30 years old (n = 14,556). For all teeth except 3^rd^ molars, probing depth (PD), and recession were recorded on six sites per tooth (mid-facial, mid-lingual, mesio-facial, mesio-lingual, disto-facial and disto-lingual). PD was recorded as the distance from the free gingival margin to the bottom of the sulcus/pocket. Recession was the distance between the free gingival margin and the cemento-enaml junction (CEJ). Recession was recorded as a negative value if the gingival margin was coronal to the CEJ. Readings were rounded to the nearest millimeter. Recession and PD measurement were summed to calculate the clinical attachment loss (CAL). Edentulous individuals and those with contributory medical history requiring prophylactic antibiotics were excluded from the periodontal examination. We also excluded participants if they had missing periodontal data, sleep data, or any of the covariates information, and performed a complete-case analysis. The final analysis included 10,291 participants.

### Definition of the exposure and the outcome

The main exposure was duration of sleep in hours based on the question how much sleep do you usually get at night on weekdays or workdays? Participants gave a range of 2–12 hours (12 was the maximum value, and was also given to those who reported more than 12 hours). We categorized sleep duration into 3 categories; 2–6 as sleep deficient, 7–8 as sleep adequate, and 9–12 as sleep excessive [27]. Self-reported sleep duration has been validated and used in the literature [28–30].

We used the case definition created by the Center for Disease Control and Prevention (CDC) and the American Academy of Periodontology (AAP) for periodontitis [31]. Periodontitis was defined as having at least 2 interproximal sites with 3 mm CAL or more and at least 2 interproximal sites with PD of 4 mm or more, that are not on the same tooth, or having at least 1 interproximal site with PD of 5 mm or more.

### Covariates

To control for confounding, we adjusted for major risk factors of periodontal disease; smoking, age, gender, race/ethnicity, education, socioeconomic level, diabetes and body mass index (BMI). In NHANES, race/ethnicity was classified as: non-Hispanic White, non-Hispanic Black, Mexican-American, other Hispanic, and other Race-including Multi-Racial. We categorized age into 30–34, 35–49, 50–64, and 65 y or older. For smoking, we used the following two question: “smoked at least 100 cigarettes in life?” and “do you now smoke cigarettes?”, and categorized participants into: ‘current smoker’ if they answered yes to both questions, ‘former smoker’ if they answered yes to the first question but no to the second question, and never smokers if they answered no to both questions. We classified participants as diabetics if they answered yes to the question “have you ever been told by a doctor that you have diabetes?”. For socioeconomic level, we used the ratio of family income to poverty, and participants were categorized into <100, 100–199, 200–399, and ≥400%, using the federal poverty level. We categorized BMI as follow: underweight (<18.5), healthy weight (18.5–24.9), overweight (25–29.9), and obese (30+ Kg/m^2^).

### Statistical analysis

Descriptive statistics for the study cohort by sleep categories were calculated. We used logistic regression models to calculate the odds of periodontitis comparing the sleep-deficient and the sleep excessive population to the sleep adequate participant (reference group). We adjusted the model for the confounding variables. We used the sample weights to account for the sampling design. Analysis was done using SAS statistical software (version 9.4; SAS Institute, Cary, NC).

## Results

The final analysis included 10,291 individuals, among whom 5,244 were periodontitis patients. Table 1 shows characteristics of the study population by categories of sleep duration. Most of the study participants were either in the sleep adequate or sleep deficient categories. The number of women who reported sleeping more than 8 hours was slightly higher than men. Most of the less than 7 hours group were in the 35–49 y age range, while most of the more than 8 hours category were older. More proportion of non-Hispanic Black participants tended to report sleep deficiency, while most of the non-Hispanic White where either in the sleep adequate or excessive categories. Most of the current smokers reported less than 7 hours of sleep. The proportion of diabetics in the excessive sleep group was somewhat higher. Higher proportion of obese were sleep deficient. Most of participants who reported 7–8 hours were in the higher socioeconomic level. Table 2 compared those with periodontitis to those with no periodontitis. About 42% of the study population had periodontitis. Periodontitis was higher among males, Mexican-American and non-Hispanic Black participants. Older individuals were more likely to have periodontitis. Never smokers were the least likely to have periodontitis, followed by former smokers; Current smokers had the highest risk. Diabetes and obesity were associated with a higher prevalence of periodontitis. Higher socioeconomic status and educational attainment were inversely associated with periodontitis. The average hours of sleep was marginally lower among participants with periodontal disease.

**Table 1:** Characteristics of the study population by sleep duration groups.

	7–8 hours (adequate)		< 7 hours (deficient)		> 8 hours (excessive)	
	N	Weighted % (SE)	N	Weighted % (SE)	n	Weighted % (SE)
**Total**	**5523**		**4077**		**691**	
**Gender**						
Male	2714	48.05 (0.81)	2047	51.54 (1.07)	312	42.00 (1.85)
Female	2809	51.95 (0.81)	2030	48.46 (1.07)	379	58.00 (1.85)
**Age (years)**						
30–34	678	11.88 (0.56)	516	13.43 (0.68)	79	12.44 (1.57)
35–49	1935	36.44 (1.13)	1550	42.44 (0.96)	167	26.61 (2.40)
50–64	1678	33.53 (1.05)	1322	31.26 (1.21)	187	27.62 (2.02)
65+	1232	18.14 (0.76)	689	12.86(0.56)	258	33.34 (2.25)
**Race/Ethnicity**						
Mexican American	819	7.99 (1.12)	551	8.32 (1.12)	93	7.49 (1.43)
Other Hispanic	525	4.98 (0.67)	459	6.58 (0.92)	51	3.87 (0.81)
Non-Hispanic White	2610	72.59 (1.79)	1445	60.83 (2.20	368	74.81 (2.44)
Non-Hispanic Black	872	7.42 (0.64)	1129	16.05 (1.36)	124	8.95 (1.39)
Other—including multi-racial	697	7.02 (0.57)	493	8.23 (0.74)	55	4.88 (0.87)
**Smoking**						
Current smoker	891	14.08 (0.59)	919	22.94 (0.91)	132	17.88 (2.11)
Former smoker	1423	27.11 (1.03)	939	24.10 (0.96)	186	26.75 (2.07)
Never smoker	3209	58.82 (0.96)	2219	52.97 (1.28)	373	55.37 (2.16)
**Diabetes**						
No	4900	91.25 (0.44)	3515	89.24 (0.63)	563	85.61 (1.68)
Yes	623	8.75 (0.44)	562	10.77 (0.63)	128	14.39 (1.68)
**Body Mass Index (kg/m2)**						
< 18.5	61	0.95 (0.17)	40	0.92 (0.19)	16	2.13 (0.84)
18.5–24.9	1525	27.62 (0.82)	960	23.21 (1.05)	195	29.46 (2.33)
25–29.9	1992	36.78 (0.93)	1379	34.20 (0.91)	213	33.81 (2.40)
30+	1945	34.65 (1.06)	1698	41.68 (0.98)	267	34.59 (2.30)
**Socioeconomic level (% FPL)**						
< 100	1369	16.20 (0.88)	1121	20.30 (1.05)	201	19.96 (2.01)
100–199	1233	16.38 (0.80)	964	19.27 (1.09)	173	18.67 (1.66)
200–399	1321	26.33 (1.21)	1004	27.51 (1.24)	162	26.60 (2.42)
400+	1600	41.09 (1.52)	988	32.91 (1.66)	155	34.77 (3.04)
**Education (years schooling)**						
< 12	1259	14.19 (0.98)	954	16.73 (0.85)	198	18.17 (1.99)
12	1111	19.20 (0.84)	948	23.20 (0.99)	157	20.61 (2.59)
> 12	3153	66.61 (1.41)	2175	60.06 (1.25)	336	61.22 (3.18)
**Periodontitis**						
No	2837	61.22 (1.45)	1898	53.80 (1.41)	312	52.84 (3.05)
Yes	2686	38.78 (1.45)	2179	46.20 (1.41)	379	47.16 (3.05)

**Table 2:** Characteristics of the study population by periodontal disease diagnosis.

	No Periodontitis		Periodontitis	
	N	Weighted % (SE)	N	Weighted % (SE)
**Total**	5047	58.02 (1.38)	5244	41.99 (1.38)
**Gender**				
Male	2021	42.25 (0.80)	3052	58.13 (0.71)
Female	3026	57.75 (0.80)	2192	41.87 (0.71)
**Age (years)**				
30–34	885	16.08 (0.70)	388	7.49 (0.52)
35–49	2146	42.95 (1.03)	1506	31.09 (1.10)
50–64	1299	29.01 (1.02)	1888	36.93 (1.17)
65+	717	11.97 (0.60)	1462	24.49 (1.03)
**Race/Ethnicity**				
Mexican American	524	5.54 (0.72)	939	11.57 (1.70)
Other Hispanic	500	4.91 (0.62)	535	6.28 (0.91)
Non-Hispanic White	2532	74.79 (1.58)	1891	59.82 (2.56)
Non-Hispanic Black	830	7.92 (0.64)	1295	14.35 (1.40)
Other—including multi-racial	661	6.84 (0.56)	584	7.98 (0.80)
**Smoking**				
Current smoker	643	11.57 (0.51)	1299	25.71 (0.79)
Former smoker	1136	24.28 (1.00)	1412	28.38 (1.05)
Never smoker	3268	64.15 (1.02)	2533	45.91 (1.00)
**Diabetes**				
No	4616	93.16 (0.45)	4362	86.01 (0.60)
Yes	431	6.84 (0.45)	882	13.99 (0.60)
**Body Mass Index (kg/m2)**				
< 18.5	52	0.84 (0.17)	65	1.27 (0.18)
18.5–24.9	1388	27.60 (0.86)	1292	24.16 (0.79)
25–29.9	1747	35.90 (0.93)	1837	35.32 (0.72)
30+	1860	35.66 (0.87)	2050	39.25 (1.02)
**Socioeconomic level (% FPL)**				
< 100	1023	13.55 (0.80)	1668	23.95 (1.15)
100–199	972	14.20 (0.83)	1398	22.22 (0.91)
200–399	1237	25.52 (1.39)	1250	28.51 (1.14)
400+	1815	46.73 (1.79)	928	25.33 (1.18)
**Education (years schooling)**				
< 12	746	9.37 (0.75)	1665	23.64 (1.06)
12	906	16.99 (0.89)	1310	25.90 (0.71)
> 12	3395	73.64 (1.23)	2269	50.46 (1.12)
**Sleep**				
Average hours		6.93 (0.02)		6.82 (0.03)

In the crude model, those who slept less than 7 hours had a statistically significant 36% higher risk of periodontitis (OR: 1.36, 95% CI: 1.23–1.50) when compared to the 7–8 hours reference group, while the odds of periodontitis for participants who slept more than 8 hours were 40% higher than the reference group (OR: 1.40, 95% CI: 1.16–1.71) (Table 3). The association was slightly attenuated in the age adjusted model. In the fully adjusted model, the odds of periodontitis among the sleep-deficient population were 19% significantly higher than the sleep-adequate participants (OR: 1.19, 95% CI: 1.06–1.38). The association among the sleep-excessive population was also attenuated and became statistically non-significant (OR: 1.16, 95% CI: 0.94–1.43).

**Table 3:** Odd ratios and 95% CI relating duration of sleep to periodontitis.

	7–8 hours (adequate)	< 7 hours (deficient)	> 8 hours (excessive)	*P*-value
**Model 1**	1.00 (ref)	1.36 (1.23–1.50)	1.41 (1.16–1.71)	<0.001
**Model 2**	1.00 (ref)	1.49 (1.35–1.65)	1.26 (1.04–1.52)	<0.001
**Model 3**	1.00 (ref)	1.19 (1.06–1.38)	1.16 (0.94–1.43)	0.01

Model 1: Crude

Model 2: Adjusted for age (30–34, 35–49, 50–64, 65+ y).

Model 3: Adjusted for age (30–34, 35–49, 50–64, 65+ y), gender (male, female), race/ethnicity (Mexican American, other Hispanic, non-Hispanic White, non-Hispanic Black, other-including multi-racial), smoking (current, former, never), diabetes (yes, no), BMI (< 18.5, 18.5–24.9, 25–29.9, 30+ kg/m2), socioeconomic level (< 100, 100–199, 200–399, 400+ % FPL), and education (< 12, 12,> 12 years of schooling).

## Discussion

The results of the present investigation showed that short sleep duration of 6 hours or less compared to sleeping the recommended 7–8 hours a night was associated with 19% increase in prevalence of periodontitis. These results are consistent with recent findings in a Turkish population in which sleep duration was found to be associated with the stage and grade of periodontitis [22]. It also supports the findings of a study that used the Taiwan National Health Insurance database and found a 36% increase in the probability of developing periodontitis among the study sample [25]. Our results are also in line with an animal study that found an increase of gingival inflammation and alveolar bone loss in sleep deprived rats. In contrast, our findings differ from results of a recent analysis by Romandini et al in which participants who slept 5 hours or less were less likely to have periodontitis [24]. The Romandini et al study used the community periodontal index (CPI) for periodontal assessment which has major limitations as it depends on few index teeth which might underestimate the prevalence of periodontitis. Furthermore, in that study, sleep duration was classified into 5 categories with the “less than 5 hours” as the reference category. This contrasts the majority of human sleep studies in which 7–8 hours, as was done in our study, were considered to be the adequate sleep duration. Lastly, the populations of the two studies are different which could explain in part the dissimilar findings.

Our findings also differ from a study by Wiener in which no significant association between sleep duration and periodontitis was found [23]. The sleep duration in Weiner’s study was dichotomized into less than 7 hours, and 7 hours or more which combine those with adequate (7–8 hours a night) and long (> 8 hour a night) sleep duration into a single category. This method of categorization fails to appreciate the U-shaped relationship between sleep and periodontitis. Long sleep duration of 9 hours or more has been shown, in several epidemiological studies to be related to an increased morbidity and mortality [32–34]. In addition, it was shown to be associated with a higher periodontitis prevalence among women and fewer remaining teeth among elderly [35, 36]. As long sleep duration might have harmful effect, combining adequate and long sleeper into a single category would probably dilute any significant results, which would explain the inconsistency in the results between the present study and Weiner’s results.

There are several plausible mechanisms to explain the observed association between short sleep duration and higher prevalence of periodontitis. Sleep is known to affect the immune system function; hence insufficient sleep could have altered the balance between the immune system and the periodontal microflora. Sleep deprivation has been linked to elevated level of pro-inflammatory cytokines that are important in regulating the inflammatory cascade such as interleukin-6 (IL-6), C-reactive protein (CRP), and tumor necrosis factor-alpha (TNF-⍺) [37–39]. Pink et al prospectively investigated the association between systemic inflammation, using fibrinogen and white blood cell counts, and periodontal disease [40]. They found systemic inflammation to be a significant predictor of periodontal tissue loss [40]; thus activation of pro-inflammatory pathways is a possible mechanism by which short sleep duration could affect periodontitis. In addition, sleep is linked to psychosocial stress and obesity which have been shown to be associated with higher periodontitis prevalence [41–43]. Since the present study was cross sectional, it could also be possible that insufficient sleep is a consequence rather than a cause of periodontitis or that the reported association between the two conditions is coincidental.

The present study has several strengths including the large representative sample size, the sophisticated sampling methods and application of the full-mouth clinical periodontal examinations protocol. Our study however has some limitations. Although research has shown that self-reported sleep duration has acceptable validity [28–30], bias due to measurement error cannot be ruled out. In addition, due to the observational nature of the study, the likelihood of confounding cannot be excluded. However, we adjusted for the established risk factors of periodontal disease which should mitigate any bias due to confounding. The cross-sectional design of our study is another limitation; Longitudinal cohort studies are warranted to confirm the findings of the present analysis, and to explore the potential causality.

In conclusion, findings of the present study showed an association between short sleep duration and higher prevalence of periodontitis in a nationally representative United States population. If these results were confirmed in longitudinal prospective studies, this might have significant health implications considering the high prevalence of periodontitis and increased number of individuals who sleep less than 7 hours, in the contemporary society.

## Supporting information

S1 Table STROBE statement—checklist of items that should be included in reports of observational studies. (DOCX)
